# Health Care Facilities Resilient to Climate Change Impacts

**DOI:** 10.3390/ijerph111213097

**Published:** 2014-12-16

**Authors:** Jaclyn Paterson, Peter Berry, Kristie Ebi, Linda Varangu

**Affiliations:** 1 Climate Change and Health Office, Health Canada, 171 Slater Street, Ottawa, ON K1A 0K9, Canada; E-Mail: peter.berry@hc-sc.gc.ca; 2 ClimAdapt LLC, 424 Tyndall Street, Los Altos, CA 94022, USA; E-Mail: krisebi@essllc.org; 3 Canadian Coalition for Green Health Care, 1724 Concession 6 West, RR #2, Branchton, ON N0B 1L0, Canada; E-Mail: linda@greenhealthcare.ca

**Keywords:** health care facility, climate change, resiliency, adaptation, extreme weather

## Abstract

Climate change will increase the frequency and magnitude of extreme weather events and create risks that will impact health care facilities. Health care facilities will need to assess climate change risks and adopt adaptive management strategies to be resilient, but guidance tools are lacking. In this study, a toolkit was developed for health care facility officials to assess the resiliency of their facility to climate change impacts. A mixed methods approach was used to develop climate change resiliency indicators to inform the development of the toolkit. The toolkit consists of a checklist for officials who work in areas of emergency management, facilities management and health care services and supply chain management, a facilitator’s guide for administering the checklist, and a resource guidebook to inform adaptation. Six health care facilities representing three provinces in Canada piloted the checklist. Senior level officials with expertise in the aforementioned areas were invited to review the checklist, provide feedback during qualitative interviews and review the final toolkit at a stakeholder workshop. The toolkit helps health care facility officials identify gaps in climate change preparedness, direct allocation of adaptation resources and inform strategic planning to increase resiliency to climate change.

## 1. Introduction

Climate change has been identified as the biggest global health threat of the 21st century [1]. In Canada, climate change is expected to pose greater health risks to Canadians from increases in the frequency and magnitude of extreme weather events (e.g., more heat events of longer duration [2], floods, droughts, wildfires andstorm surges), poor air quality, impacts on drinking and recreational water quality, food-borne diseases and vector-borne and zoonotic diseases [2,3]. Health care facilities play a critical role in reducing health impacts from climate change by treating illnesses and injuries attributable, at least in part, to climate-related hazards, caring for patients during and after disasters [4] and actively participating in community efforts to adapt to and mitigate climate change [5]. Health care pressures in communities will increase with climate change and are expected to occur coincident with important socio-economic changes (e.g., ageing population, increased urbanization) and environmental degradation that can further increase health risks in populations and demands on health care facilities [6]. 

A changing climate threatens the quality and continuity of care provided at health care facilities due to more frequent and severe extreme weather events and increased health risks from a range of other climate hazards including food-, water-, vector-borne and zoonotic diseases and poor air quality. Health care facilities will become increasingly vulnerable to impacts from climate change without adaptation [2]. For example, extreme weather events can lead to public health emergencies which can overwhelm health care facility capacity, disrupt services, and damage facility infrastructure which can subsequently pose health risks to patients and staff [4,7]. In many regions, more extreme heat events and generally warmer summers are expected to increase heat-related illnesses and exacerbate chronic diseases [1,8]. Studies have linked extreme heat events with increased health care facility visits [9,10] and similar correlations have been made with poor air quality days [11,12]. Health care facilities are highly dependent on critical community services (e.g., electricity, clean drinking water, food service delivery, waste disposal and treatment) that are vulnerable to power disruptions as evidenced by recent events in Canada [13] and internationally [14,15]. Health care facility officials may have a false sense of security that critical services will be available in emergencies which may not be the case with changing weather patterns due to climate change [14,16]. Risks of critical infrastructure loss from flooding events are expected to rise with climate change and health care facilities will need to adapt systems accordingly. Health professionals will need to be better trained and equipped to diagnose and treat new and emerging diseases and to respond to a wider range of climate-related public health emergencies. Costs and unforeseen expenses associated with climate change impacts may exacerbate existing financial challenges and strain functional capacity of health care systems at the community level [17].

Health care facilities in developed nations, like Canada, are not immune to climate change impacts. Recent climate-related disasters in Canada, the United States, Australia and Europe have highlighted challenges in disaster preparedness associated with climate hazards. The Alberta floods (2013), Hurricane Sandy (2013), Hurricane Juan in Nova Scotia (2003), Hurricane Katrina (2002), and the ice storm in Eastern Canada (1998) created unexpected challenges for health care facilities that exceeded coping capacity in some cases. Challenges included damage to infrastructures, limited access to essential services, increased patient loads, and issues with maintaining supply chains, such as essential drugs. The 1998 ice storm led to widespread power outages and surges of patients at hospitals with a wide range of injuries and acute illnesses such as fractures, hemorrhages, hypothermia, and carbon monoxide poisoning. Many health facilities were called upon to provide shelter for persons without power at home and critical supplies to other hospitals [18]. Some studies have highlighted the need to bolster health care facility emergency preparedness in Canada [19,20,21], further highlighting the need to improve disaster resiliency to prepare for a changing climate. Knowledge of the impacts of climate change on the health care sector is increasing [1,22,23]; however, guidance for developing comprehensive adaptation strategies for health systems is needed [4,24].

Information exists to support efforts by health care facilities to prepare for climate change but guidance is piecemeal. For example, Blashki provides examples of adaptations to climate change hazards for health systems that are included in Australia’s National Adaptation Research Plan [24]. Hiete *et al*. provide strategies to reduce a health care facility’s vulnerability to power outages and VanVactor outlines recommendations for ensuring supply chain management processes are resilient in emergencies [16,25]. Health care facilities contribute significantly to greenhouse gas emissions through the energy intensive 24 h operation of services and to environmental degradation through the high demand of health care services and operations on natural resources (energy, water, and food procurement) [26,27]. As such, tools are available to help health care facilities reduce their environmental footprint [5,26,28]. A lack of a coherent and integrated approach to adaptation results in preparedness gaps that can leave some health care facilities and surrounding communities vulnerable. Furthermore, no climate change resiliency tool has been developed and tested in Canada and tailored to the needs of facilities in the country.

Health care facilities need a comprehensive tool to support climate change resiliency. This paper describes the methods undertaken to produce a climate change resiliency assessment toolkit for use by health care facility officials. The toolkit consists of: (1) a checklist for officials who work in areas of emergency management, facilities management and health care services and supply chain management; (2) a facilitator’s guide for administering the checklist; and (3) a resource guidebook to inform adaptation. The facilitator’s guide provides an overview of climate change impacts on health and on health care facilities, information for officials on how to complete the assessment checklist and suggestions on how to adapt using the assessment results and information from the resource guidebook. A project lead within the health care facility can use the facilitator’s guide to aid officials in completing the checklist and in identification of needed adaptation strategies. The methodology used to develop the toolkit, a description of the resiliency indicators used to develop the checklist and recommendations for operationalization of the toolkit in Canada and internationally are discussed below.

## 2. Methods 

A mixed methods approach was used to develop the climate change resiliency assessment toolkit for health care facilities. Methods included a literature review to identify indicators of health care facility resiliency, development and pilot testing of an assessment checklist, and a workshop of key stakeholders to validate the toolkit and identify optimal strategies for its dissemination and uptake in health care facilities.

### 2.1. Literature Review of Health Care Resiliency Indicators

A literature review to identify indicators of health care facility resiliency to climate change was conducted in September and October 2012 and used to inform development of the assessment checklist. Peer-reviewed and grey literature published between 1 January 2003 and October 2012 was retrieved using a standard search strategy based on keywords related to:

- Community and health care facility resilience to health impacts from climate change
- Climate change and health hazard mitigation and prevention
- Management of climate-related disasters or emergencies
- Sustainable health care practices that reduce climate-related health burdens
- Communication of heat-health and other climate-related health risks to patients and to the public

Separate searches were conducted to identify relevant articles describing resiliency in terms of emergency management and environmental sustainability. Keywords were used in the literature searches to capture the climate-related stimulus (e.g., climate change, global warming, extreme weather, heat, cold, air pollution, ultraviolet radiation, wildfires, flooding, storms, drought, water-, food-, vector-, rodent-borne disease), entity impacted or impact type (e.g., hospital, health care facility, food service, supply chain, health care, morbidity, mortality, disease, infrastructure damage), emergency management (e.g. plan, evacuation, prevention, recovery, response) and/or environmental sustainability (e.g., greening, green, recycle, reduce, reuse, waste management). The following databases were searched: EMBASE, Global Health and Ovid Medline. A total of 2,342 peer-reviewed journal articles were identified and titles and abstracts were scanned for relevance. Articles were retrieved and further included for analysis if they were written in English and had information pertinent to the Canadian context (developed countries). Articles that did not directly address indicators of health care resiliency to climate change were excluded, with 34 peer reviewed articles remaining. In addition, a total of 30 grey literature reports were included, which were retrieved from various government (e.g., World Health Organization, Department of Health in the United Kingdom, Health Canada) and non-government websites (e.g., Health Care Without Harm, Centre for Excellence in Emergency Preparedness). The sixty-four references included in this study that informed development of the assessment checklist can be found in the Supplementary Materials.

### 2.2. Indicator Selection and Toolkit Development

Resilience refers to the “capability of individuals and systems to cope with significant adversity or stress in ways that are effective and that tend to result in an increased ability to constructively respond to future adversity” [29]. Based upon this definition, indicators were identified to capture key climate change resiliency metrics to describe actions to prepare for, cope with and constructively respond to extreme weather events and gradual shifts in average weather conditions. Best practices identified from the literature review helped inform selection of the indicators used to develop the assessment checklist. Indicators were categorized into two general groups that included emergency management and strengthening health care services and climate-proofing and greening operations (referring to measures to reduce greenhouse gas emissions, reduce waste and improve the health of the environment).

Emergency management indicators to evaluate and assess the status of health care services and emergency management were further grouped based on the key exposures and health outcomes expected to pose increasing risks to the operation of, or populations served by these facilities [30,31]. Effectively addressing the health risks of climate change requires preparations for critical emergencies and for events that extend in duration (e.g., drought, disease outbreaks). The indicators for emergency management and strengthening health care services are comprehensive and capture requirements for health care facilities to prepare for and respond to a range of situations that may result from climate change and increase health risks.

The indicators were assessed by an expert advisory committee and used to develop the resiliency assessment checklist for health care facilities. Members of the advisory committee included emergency management and facilities management officials from select hospitals in Canada, and representatives from the Canadian Coalition for Green Health Care, the United Kingdom’s National Health Services Environmental Sustainability Branch, the Departments of Environment and of Health and Wellness in Nova Scotia. The final set of indicators used to develop the assessment checklist in the toolkit is presented in Table 1.

The checklist was designed for use by health care officials within the health care setting to obtain data on the status of current activities that convey resilience to current climate variability and on activities that help prepare for future climate change impacts. Once completed, it provides information that can be mainstreamed into regular planning and program decision making. Each indicator in the table was used to develop one or more corresponding questions in the assessment checklist. For most questions, response options included “Yes”, “No”, “Somewhat, Sometimes”, “I don’t know” or “Not Applicable”. The first iteration of the toolkit consisted of a two page backgrounder and a list of 92 checklist questions organized into three areas including Emergency Management (n = 45 questions), Facilities Management (n = 26 questions) and Health Care Services and Supply Chain Management (n = 21 questions). 

**Table 1:** Climate change resiliency indicators for health care facilities.

General Resiliency Indicators	
Assesses cost-effectiveness of health care facility adaptation to climate change by quantifying the benefits and costs of implementing new or improved measures to address risks Capitalizes on opportunities to learn and increase awareness about climate change, its impacts and co-benefits of sustainable practices Builds and enhances climate change knowledge capacity as it relates to hazards of concern for the health care facility Ensures adequate leadership and allocation of staff roles and responsibilities in efforts to increase resiliency Builds climate change adaptive capacity through partnerships and by securing mutual support	
**Emergency Management and Strengthening Health Care Services**	
**General**	Assesses health risks to staff, patients and visitors from climate-related hazards of concern including assessments of the effectiveness of existing control measures Establishes plans specifying how the facility will manage staff-related issues during an emergency (e.g., when staff are affected while at work, when staff are unable to come to work) Secures access to critical back-up supplies and resources (medical equipment, treatment supplies, required experts, alternative energy supplies) Ensures sufficient emergency room surge capacity to manage climate-related emergencies and disasters (e.g., extreme heat event) effectively Ensures the allocation of resources for mitigating and preventing climate change impacts of extreme weather/climate events As part of the emergency plan, adopts an incident management system, rapid needs assessments and implementation of incident response plans that are robust in the face of more severe and frequent climate-related emergencies Ensures that coordination and communication mechanisms are in place with external agencies and stakeholders Raises awareness of health care facility staff, patients, visitors and the community of risks to health from climate-related hazards and effective health protection measures Develops systems for monitoring injuries and diseases from climate-related hazards including monitoring health outcomes to vulnerable patients (e.g., elderly, immobile, infants, critical care patients) in the event of a climate-related emergency or disaster
**Extreme Weather**	Establishes mutual aid/assistance agreements (mutual aid, transfer of patients, sharing of resources and supplies) with other institutions during response and recovery from an extreme weather event or natural disaster Ensures emergency plans for extreme weather events are consistent with community plans and updated regularly and iteratively based on new information on how climate and vulnerability affect risks Provides psychological first aid to address mental health impacts of emergencies and disasters on patients, health care facility staff and visitors Develops systems to act upon extreme weather advisories and warnings to reduce health risks Undertakes ongoing evaluations of climate change impact response and recovery protocols as they relate to extreme weather and climate events to identify what can be improved Provides training and exercises for preparing for, responding to and recovering from extreme weather-related emergencies including the testing of back-up emergency power sources
**Food-borne Diseases and Outbreaks**	Establishes protocols for timely diagnoses, treatment and reporting of food-borne disease outbreaks, including climate-related diseases Establishes protocols for the health care facility food service to respond and recover from an extreme weather event (e.g., emergency menus) and food-borne outbreaks (sanitation, disinfection, isolation) Ensures patient identification during an emergency or outbreak to direct food delivery to patients Ensures that staff who oversee food services are aware of new climate-related risks and incorporate them into planning and preparedness processes Stores adequate back-up food supplies that will provide enough food during an emergency or outbreak Develops systems to act upon water-borne contamination advisories and warnings Ensures that kitchens have adequate food storage maintenance and temperature control measures in place and are robust in emergencies Diversifies sustainable food service providers and secures access to essential backup food sources via multiple memoranda of agreement with different vendors and through cooperative agreements with other health care facilities Monitors food resources during emergencies to ensure adequate supplies throughout the duration of the event and ensures protocols are in place to guide the rationing of limited food supplies Implements response plans that include regular communication to food service staff and the identification of alternate food production areas Ensures food service staff adopt proper storage and sanitary food handling and cooking practices and minimize time between food preparation and consumption Increases awareness of food laws related to food systems and the potential risks of contamination from its source to delivery, including under future climate change conditions Establishes mechanisms to identify and incorporate new risks to the food supply from climate change
**Infectious Diseases**	Ensures infection control practices are in place (screening, vaccination, sanitation, isolation, use of personal protection equipment, disinfection, notifications to staff, patients and visitors, waste management) and areroutinely observed Develops outbreak management plans and evaluates climate change preparedness, response and recovery protocols as they relate to infectious diseases Participates in educational activities to increase knowledge of risks from new and emerging infectious diseases including the potential impacts of climate change Ensures infection control practices and mitigation measures are integrated into building design, premises, HVAC and filter maintenance (e.g., ventilation systems) Conducts infection proofing of existing buildings and premises (hand washing, safe water supply, ventilation, isolation rooms and patient flow design to minimize infection transmission) Reports communicable diseases as mandated by health legislation Secures access to necessary treatments and supplies to manage infectious disease outbreaks including zoonotic and vector-borne diseases Collaborates with public health department(s) and others to reduce vector breeding sites (e.g., pools of water) on facility property Establishes protocols for timely diagnoses, treatment and reporting of infectious disease outbreaks, including zoonotic and vector-borne diseases Links infectious disease surveillance and early warning systems with the public health department Trains health care staff on how to respond to new health threats, post-disaster case management and proper infection control
**Air Pollution**	Implements strategies to notify health facility staff, patients and visitors of air pollution advisories and warnings (e.g., smog, wildfire smoke) Supports work-from-home and telehealth policies, when possible, to reduce staff exposure to air contaminants Ensures rapid clean-up and recovery from extreme weather events to avoid indoor air quality problems (e.g., mould growth associated with flooding) Adopts plans or protocols to minimize increased health burdens during smog events or poor air quality days, including measures to ensure vulnerable staff and patients minimize time outside
**Water-borne diseases and outbreaks**	Secures access to essential backup water supplies to respond to and recover from water-related emergencies Ensures effective and timely delivery of safe water during emergencies over the short- and long-term Establishes protocols for timely diagnoses, treatment and reporting of water-borne illness cases Establishes protocols for decontaminating water (e.g., boil water advisories and subsequent instruction) Ensures kitchens have adequate supplies of clean and potable water Monitors water resources during emergencies to ensure adequate supplies throughout the duration of the event and ensures protocols are in place to guide the rationing of limited suppliesEnsures maintenance and regular cleaning of water storage and distribution systems
**Climate Proofing and Greening Operations**	
Conducts assessments of energy use and of cost-effective measures to reduce energy consumption Contributes to climate change adaptation or greenhouse gas mitigation by implementing sustainable practices, including energy use monitoring, diversifying energy sources, greening fleet vehicles, and installing energy efficient heating, ventilation and air-conditioning systems Adopts climate change resilient health care facility infrastructure and design Adopts and supports mechanisms to filter indoor and ambient air pollutants, including the establishment of greenspaces Minimizes the release of harmful air pollutants through facility operations Implements or adopts mechanisms for proper waste management from generation, segregation, collection, transportation, storage, treatment to final disposal of medical equipment, supplies and linen Supports waste management practices that reduce exposure to food contamination Considers food sustainability practices such as monitoring greenhouse emissions of food vendors and suppliers and community food security indicators Supports initiatives to increase awareness of the co-benefits of active transportation, measures to reduce air pollution and proper waste management practices among health care facility staff, patients and visitors Sets water usereduction targets and develops water conservation strategies	
**Climate Proofing and Greening Operations**	
Regularly monitors water-use Contributes to climate change adaptation and greenhouse gas mitigation through the procurement of sustainable products and supplies Increases awareness of sustainable food system alternatives with climate change greenhouse gas mitigation and adaptation co-benefits Procures health care facility foods from local sources and builds capacity by establishing partnerships with food system community partners and local vendors in support of community food security (including food safety and reduced reliance on long distance travel of food)	

### 2.3. Pilot Testing and Expert Review of the Assessment Checklist

The checklist was reviewed and revised based on input from the expert advisory committee before being further tested at health care facilities. To ensure that the checklist would meet the needs of facilities in Canada in terms of relevance, understandability, and ease of use, it was piloted in six health care facilities representing three provinces—Nova Scotia, Ontario and Manitoba. The pilots were chosen based on the following criteria: facilities representing different provincial health care systems in Canada; facilities of different sizes (indicated by the number of beds); and facilities faced with a wide variety of climate change hazards (e.g., sea level rise, drought, flooding, hurricanes and infectious disease outbreaks). Pilot testing of the checklist was conducted from 4–8 February 2013 through expert interviews to inform revisions. For each facility, one facility official was responsible for consulting with relevant officials to complete the checklist prior to interviews. Interviewees for each facility included all individuals who contributed to answering the checklist questions. On the dates of the interviews, group discussions were held among a member of the research team and health care facility officials. The interview questions posed are presented in Table 2. Interviews were recorded and responses were analyzed to identify needed revisions.

**Table 2:** Interview questions to pilot health care facility officials.

Do you think the tool is useful? Does the tool adequately assess resiliency? If yes, why and how? If no, why and what should change to better assess resiliency? Does the tool provide valuable information about risks and risk management that builds on existing risk management framework(s) you currently use? Would you use this tool to identify gaps and to inform future planning or activities? If yes, how should the tool be packaged and presented for effective use? If no, why and what would need to change to make the tool more valuable? In considering “resiliency”, are these the right sections? *Question Prompts:* Do they facilitate mainstreaming of climate change information into regular activities? Did you find that most or all of the questions informed areas where more action may be needed to increase your resiliency to climate change? Did you find that questions highlighted information gaps? (e.g., if you answered ‘I don’t know’) Were you always confident in providing a response or would you have preferred a legend or more objective criteria from which to guide your responses? Did you have enough knowledge to answer the questions with confidence? Did any of the questions strike you as unnecessary or contribute little value? Do you think that any questions were too similar/repetitive? Are the questions detailed enough to provide value to your planning and activities aside from regular risk management activities and to enhance resiliency? Is this checklist too long or too short?

After revisions were made based upon interview results, a stakeholder workshop was held on 20 March 2013 to present and validate the final toolkit.

## 3. Results 

### 3.1. Summary of Findings from the Literature Review that Supported Indicator Development 

Efforts to enhance emergency management systems, strengthen health care services and actions aimed at climate-proofing and greening operations can help build resiliency of health care facilities to climate change impacts [4,7,25,32,33]. Effective practices as they pertain to these areas are described below.

#### 3.1.1. Emergency Management and Strengthening Health Care Services

Robust emergency management and health care strengthening programs that include measures to prevent, prepare for, respond to and recover from extreme weather events or public health emergencies increase resilience to climate change [31,32,34]. Standard emergency management approaches, such as all-hazards programs need to be modified to incorporate observed and projected alterations in the frequency, intensity and duration of extreme events to increase preparedness [32]. Managing risks from climate change (such as extreme weather) requires health care facilities to regularly undertake risk assessments that integrate current climate variability and future climate change [24] to support effective forward planning [31,35]. In regions found to be vulnerable to climate hazards such as floods or storm surges, adaptation efforts to increase the safety of buildings and protection of critical equipment can increase resilience [24] as can efforts to prepare for patient surges and associated pressures. 

To anticipate increased health care demands and prepare accordingly, information on climate change and health vulnerabilities at the community level can be used [2]. In many urban communities in Canada, climate change will contribute to poor air quality (e.g., from wildfire smoke, smog, allergens) that can lead to increased hospital cases of asthma, COPD, acute bronchitis, chest pain and other cardio-respiratory illnesses [3,36]. Early warning systems can help increase preparedness to support the delivery of care. Health care facilities can benefit by being linked to weather alert and warning systems [37] such as heat alert and response systems (HARS) [34]; air quality alerts [38]; boiled water advisories and critical service disruption notifications to better anticipate increases in hospital visits. Many communities in Canada have adopted HARS to notify vulnerable groups, the general public and health care and community services of impending extreme heat events and to provide information on how people can protect themselves from heat-health impacts [39]. Such systems can help health care facilities be more prepared for increased heat-related hospital visits.

Increasing mortality from heatwaves and other extreme weather events, changes in the geographic range and incidence of climate-sensitive infectious diseases, increasing morbidity and mortality from poor air quality and other health impacts will require modification of current public health programs and training of health care providers to be prepared for patients presenting with currently unexpected climate-sensitive health outcomes [40]. As such, training programs could be enhanced to ensure adequate protection and care of patients with specialized needs (e.g., infants and children) [41]. Furthermore, information on range expansion of specific diseases acquired through national and regional assessments can be used to inform rapid testing, diagnosis, treatment and surveillance of diseases to protect health [24]. Climate-induced range expansion of the deer tick that harbors Lyme disease is occurring rapidly in Canada [42,43,44] and warmer weather and more precipitation events may increase the abundance of mosquito species that carry West Nile Virus [45,46].

Once health care facilities have a better understanding of risks in their area, officials can create new or enhance existing protocols to improve risk management. For example, health care facilities in areas vulnerable to extreme heat should develop plans and protocols to guide actions that need to be taken during an extreme heat event (see Table 3) [39].

**Table 3:** Health care facility protocol for ensuring the health and safety of patients during extreme heat events.

○ Develop emergency surge procedure to ensure adequate capacity and personnel ○ Protect staff from heat illness and provide adequate work rest cycles ○ Follow regional requirements to keep the facility cool and comfortable ○ Evacuate rooms if extremely high temperature occurs (case by case basis) ○ Ensure cooling supplies are available for your patients and residents ○ Provide cooling areas ○ Keep medications cool ○ Ensure meals have high water content and that spoiled food is discarded ○ Review clinical management of patients and residents most at risk either due to reduced mobility, chronic illnesses, use of certain medications, social isolation, inadequate housing or environmental factors ○ Increase frequency of patient observations, especially those at high risk, and advise staff to closely monitor early indicators of heat illnesses and initiate appropriate treatment ○ Consider outdoor temperatures when planning group activities

Logisticians should be cognizant that more extreme weather events can increase risks of flooding at supply warehouses, damage to storage facilities, and disruption of communication lines and transportation [25] and therefore diversify suppliers and develop plans accordingly. Emergency preparedness programs could also enhance contingency plans for timely delivery of medical supplies and establish mutual aid agreements with other health care facilities to be resilient in emergencies where resources are limited [33].

Efforts to support community resilience to climate change should be implemented in addition to regular health care facility risk management processes. Some communities are undertaking climate change and health vulnerability assessments [47]; health care facility officials can participate in these efforts to support the identification of community level vulnerabilities and contribute to better estimates of health system adaptive capacity. Health care facilities also could participate in disaster risk management exercises to promote coordination and collaboration during a real event. Real time reporting of climate-sensitive health outcomes can help public health agencies take action to prevent public health emergencies [48,49]. Syndromic surveillance of health outcomes attributable to extreme heat or other climate hazards using hospital visits or other health information systems (e.g., health help lines, clinician visits) can support public health programs such as monitoring and providing early warning of impacts, informing and improving the public health response to climate hazards. It also provides a source of morbidity data to use in the process of continually assessing, evaluating and improving health programs [18,49]. As one example, the British Columbia Centre for Disease Control is involved in efforts to link health care facility syndromic surveillance with air quality issues from wildfire smoke and extreme heat events. The system will better equip public health officials with information needed to target key vulnerable groups.

#### 3.1.2. Climate-Proofing and Greening Operations

Most infrastructure was built under the assumption that the weather in the next few decades will be similar to the weather today. As a result, some critical infrastructure, such as health care facilities are located in flood plains and along coastal regions that are at risk of inundation [40]. As such, health care facilities may need to take steps to make infrastructure more resilient to climate change. Robust facility structural elements (e.g., roofs, doors, windows) and non-structural components (e.g., computers, diagnostic equipment, HVAC systems, back-up generators) need to be able to withstand extreme weather events that may cause acute or gradual damage, and/or may need to be moved above possible floodwaters. Regular maintenance, prioritizing replacement of old equipment and use of new and emerging technologies to mitigate the effects of such events can reduce vulnerability [14,50]. For example, health care facilities in drought prone regions that may be vulnerable to future water shortages can install storm water collection systems, design landscapes with drought resistant native plants, and conserve water by using appropriate low flow taps, nozzles and toilets. Health care facilities can increase their resiliency to extreme heat events by installing devices and equipment for monitoring indoor temperatures, cooling existing buildings and outdoor spaces, blocking direct sun, and increasing air flow and reducing humidity [39,50]. In addition, encouraging sustainability practices, such as turning off equipment when not in use, has shown to be a cost effective [28].

Energy supply systems in many communities are vulnerable to more frequent and severe extreme weather events and other pressures such as increased demand from population growth. Back-up power sources and strategies to deal with long lasting electricity disruptions will better prepare health care facilities for emergencies and disasters. In addition to disrupting patient care, power-outages can affect patient identification and records, access to clinical care, the demand for services, communication systems, and transportation to and from the health care facilities [14,16] and increase risks of infectious diseases [51]. Back-up generators should be tested and protected to ensure they are functional in an extreme weather event [35] and use of combined heat and power systems as a replacement for back-up generators should be evaluated. In cases where power is limited during disruptions, electricity should be re-directed to clinical areas to meet needs of vulnerable patients [14]. Plans and protocols for managing outcomes of power-outages, such as mandatory evacuation, should be developed, reviewed, and tested regularly to ensure that effective measures are in place to continue to provide high quality health care when electricity is limited.

Greening initiatives which invest in energy efficient solutions can help mitigate greenhouse gas emissions, and reduce health care facility costs and vulnerability to power disruptions [52,53]. Health care facilities can improve energy efficiency and save energy costs by using low energy lighting, boilers and electrical systems, increasing reliance on natural ventilation, promoting active transportation and the use of energy efficient vehicles, providing distant counseling services and using green procurement (e.g., purchasing local food) [5,6,7,24,54]. E-health, which includes electronic methods of information exchange among health care professionals and patients (e.g., use of email delivery of diagnostic results), can help reduce greenhouse gas emissions from patient commuters; however, backup plans should be developed to address unreliable power or energy supplies [55]. Greening health care facility properties (e.g., planting trees, grass and gardens) offers multiple health co-benefits such as the provision of natural shade for patients, staff and visitors during extreme heat events, reduced risk of facility flooding through the creation of natural flood water infiltration and the improvement of air quality by filtering pollutants [28]. Installation of high performance windows and exceeding minimum insulation requirements can also improve energy efficiency and enhance the comfort and safety of patients during extreme temperature events [56].

### 3.2. Results from the Interviews

Checklists were completed for all pilot health care facilities and interview respondents provided feedback based on questions posed during interviews. Health care facility officials indicated that the tool was useful, particularly in informing where efforts were needed to strengthen emergency management and health care services activities and partnerships, make infrastructure more robust and increase awareness of the need to be more sustainable. Because many of the questions focused on emergency management and health care services activities that are already standard practice, it was suggested that the tool may be more useful for health care facilities with fewer resources or those where officials have less experience with major events or emergencies. There was consensus that the checklist reinforces the need for robust emergency management measures in a changing climate. Interviewees noted that the checklist could be valuable component to existing risk management processes (e.g., enhancing existing Hazard Risk Vulnerability Assessment tools). The checklist was useful in helping to identify risks from gradual weather changes such as degradation of infrastructure and an increasing number of people vulnerable to climate-related impacts and in need of health care (e.g., ageing population combined with greater exposure to climate-related hazards).

Interviewees stated that the checklist is a helpful resource to inform planning. By revealing preparedness gaps and areas where improvement may be needed, the checklist provides evidence for action to reduce current and future climate-related risks. As one example, interviewees indicated that health care facilities use off site warehouses for supply storage. The location and number of warehouses may change over time as well as transportation routes to access supplies. These factors should be taken into account during contingency planning since more extreme weather could damage warehouses or reduce access to supplies during extreme weather. Some mentioned that the facility will likely need to move back-up generators or install new chillers and cooling towers. Respondents indicated that the checklist helps identify the types of emergency responses that may become more common in a changing climate; for example, there may be more evacuations needed with increased floods. The checklist raised awareness of potential cost-savings actions such as installation of new energy efficient systems, water conservation methods and better infrastructure that can withstand extreme weather. A few questions in the assessment checklist inquired about the status of surveillance and monitoring of climate-attributable health outcomes. Respondents indicated that this is a lead responsibility of public health departments but that health care facilities could support surveillance and monitoring activities if resources and guidance were available.

Interviewees provided a number of recommendations to improve the tool. Many respondents suggested that information on climate change risks in different regions of Canada and implications for health care facilities be provided as context prior to completing the checklist. Many respondents indicated that the checklist does a good job in identifying gaps and needs, but that more information is needed on how to be more resilient. A best practices resource was suggested as one way to help inform resiliency measures as well as sharing lessons learned with other health care facilities. Those who were in charge of completing the assessment checklist indicated that it took a long time to fill out because many different people needed to be consulted. As a solution, it was suggested that the checklist be completed as a group of knowledgeable individuals with senior level responsibility in managing emergencies, facility operations (e.g., HVAC systems), supply chain management and health care services and that one person facilitate the group meeting. It was suggested also that the checklist be available online and formatted to include scoring. This would help speed up the process of filling out the checklist and enable progress to be tracked over time. It was recommended that a comments field should be included after each question to provide further information as needed. Interviewees noted that it will be challenging to get resources for improvements that may be needed. As such, it was suggested that preparing for climate change and providing access to resources be part of health care facility standards nationally or provincially. Table 4 lists some of the key recommendations from respondents.

**Table 4:** Suggestions from interviewees on how to improve the tool.

**Improving Value of the Tool** Shorten the tool by eliminating repetitive questions Provide more detail to increase value of some questions Make questions less wordy and more concise Include comments field after each question Include column to indicate if other stakeholders have primary role Re-structure the tool to improve categorization of topics Distinguish between current and future risks Distinguish between acute impacts and gradual impacts Include best practicesInclude scenarios to provide more context on climate impacts
**Packaging the Tool** Promote use of the tool as a complimentary exercise to build on regular management activities Improve information about how the tool will benefit stakeholders Make the tool available electronically and include scoring Include resources and additional tools to help fill identified gaps Consider including a power-point presentation as a facilitator’s guide Ensure that objectives are clear (*i.e*., intrinsic use only, intended use for planning) Present tool as a team exercise and suggest one lead to facilitate the process of completing the checklist Indicate that the tool is a working document and can be an iterative process in the long-term Ensure tool is applicable to hospitals, health authorities and other government stakeholders Consider expanding applicability to other health care institutions (e.g., senior care homes)

### 3.3. Description of the Final Health Care Facility Toolkit 

The health care facility resiliency toolkit includes an assessment checklist, a facilitator’s guide and a resource guidebook. The checklist has 82 questions within four broad areas: general information (n = 4), assessing climate-related risks (n = 19), risk management (n = 45) and building capacity to adapt to climate change (n = 14). The checklist provides a qualitative assessment of resiliency by posing questions with possible responses of “Yes”, “No”, “Somewhat, Sometimes”, “I don’t know” or “Not Applicable”. All questions include a comments box where other pertinent information, such as gaps in knowledge and data, roles and responsibilities and status of activities can be recorded and updated during future assessments. The facilitator’s guide and the resource guide were developed in response to feedback received during pilot testing of the draft toolkit. The facilitator’s guide provides background information on climate change risks to health care facilities in Canada and instructions for health care facility officials to aid in completion of the checklist. The resource guidebook includes information highlighting effective measures that can be taken to reduce risks to the facility from climate change. The toolkit is available for download at http://greenhealthcare.ca/climateresilienthealthcare/.

The resource guidebook provides helpful resources for health care facilities to increase understanding of climate change and health risks and actions to increase resiliency. In Canada, resources are available to increase awareness of climate change risks to the health care sector. In 2008, Health Canada released a report entitled: “*Human Health in a Changing Climate: A Canadian Assessment of Vulnerabilities and Adaptive Capacity*” which provides information for Canadians and health authorities on climate change and health risks as well as the adaptive capacity of the health sector in Canada to manage risks [2]. A Government of Canada climate change impacts and adaptation assessment, released in 2014, provides updated information on climate change and health challenges in urban, rural, coastal and northern communities and innovative adaptation measures available to health sector decision makers [3]. The Canadian Coalition for Green Health Care provides information to help hospital officials reduce the environmental footprint of their facility and mitigate greenhouse gases [56]. Internationally, the Intergovernmental Panel on Climate Change produces regular assessment reports [57] and special reports (e.g., Managing the Risks of Extreme Events and Disasters to Advance Climate Change Adaptation: a Special Report of the Intergovernmental Panel on Climate Change) that includes information on climate change risks to human health. The WHO has published information about climate change and health related risks for a number of areas including extreme heat [58], flooding, economic valuation, gender implications [59] and innovative ways to reduce risks, for example, by linking climate services with emergency management and health care services [60]. Links to these resources and others are provided in the resource guidebook to facilitate its application and subsequent use to increase climate resiliency in health care facilities. Completion of the assessment checklist by a health care facility provides information on the degree to which that facility is resilient to current climate variability and future climate change based upon the indicators presented above. Identification of important gaps related to emergency management and health care services, greening of operations or supply procurement and management may be used by hospital officials to inform the implementation of needed adaptations.

## 4. Conclusions

This study contributes to a growing evidence base of practical information to help health care facilities adapt to climate change. Application of the health care facility resiliency assessment toolkit can supplement existing hazard identification and assessment processes, support the development and enhancement of emergency management and health care services programs and support engagement of partners in efforts to address climate change risks and build community resilience. The toolkit also highlights the importance of investing in environmentally sustainable measures to help mitigate greenhouse gas emissions, improve the state of the environment and human health.

Future efforts should be made to develop a more comprehensive best practices guidebook and incorporate a scoring component to enable facility officials to track progress overtime. Similar tools could also be developed for smaller or more specialized health care facilities as needed.

## Supplementary Files

Supplementary File 1
