# Supplementary material for: Health Care Facilities Resilient to Climate Change Impacts
Source: Int J Environ Res Public Health. 2014 Dec 16;11(12):13097–116. doi: 10.3390/ijerph111213097 (PMC4276665; doi:10.3390/ijerph111213097)
Supplement: Supplementary File 1 [file ijerph-11-13097-s001.pdf]

Supplementary Material

Health Care Facilities Resilient to Climate Change Impacts

Sixty-Four References Included in This Study to Inform the Development of the Assessment Checklist

1. Alt, S. Think of environment when choosing supplies. *Hosp. Mater. Manag.* **2001**, *26*. Available online: http://www.proquest.com/ (accessed on 16 October 2012).
2. Becker, C. Going to waste: One surgeon's trash can be a hospital's treasure as providers figure out how to minimize opening supplies that don't get used. *Mod. Healthc.* **2002**, *32*, 22–24.
3. Bednar, B. Using green bricks and mortar for dialysis clinic construction. *Nephrol. News Issues* **2011**, *25*, 29–31.
4. Benko, L. Not so golden now: California hospitals scramble to keep energy use low as rate hikes bite hard. *Mod. Healthc.* **2001**, *31*, 20–21.
5. Bezzant, A. Nurses initiative saves water while saving lives. *Aust. Nurs. J.* **2007**, *16*. Available online: http://search.informit.com.au/browseJournalTitle;res=IELHEA;issn=1320-3185 (accessed on 1 October 2012).
6. Boxall, A.; Hardy, A.; Beulke, S.; Boucard, T,; Burgin, L.; Falloon, P.D.; Haygarth, P.M.; Hutchinson, T.; Kovats, R.S.; Leonardi, G.; *et al.* Impacts of climate change on indirect human exposure to pathogens and chemicals from agriculture. *Environ. Health Perspect.* **2009**, *117*, 508–514.
7. Canadian Coalition for Green Health Care (CCGHC). Building green hospitals checklist. *Healthc. Financ. Manag.* **2008**, *62*, 93–95.
8. Canadian Medical Association Editorial. The Kyoto Protocol: In force? *Can. Med. Assoc. J.* **2005**, *172*, doi:10.1503/cmaj.050083.
9. Canadian Committee on Antibiotic Resistance. Infection Prevention and Control Best Practices for Long-Term Care, Home and Community Care Including Health Care Offices and Ambulatory Clinics. Available online: http://www.phac-aspc.gc.ca/amr-ram/ipcbp-pepci/pdf/amrram-eng.pdf (accessed on 10 November 2012).
10. Canadian Nurses Association (CNA). The Environment and Health: An Introduction for Nurses. Available online: http://www.cna-aiic.ca/en/on-theissues/environmental-health/environmental-health-principles/ (accessed on 27 August 2012).
11. Centre for Sustainable Healthcare (CSH). Green Nephrology Summit, London England. Available online: http://sustainablehealthcare.org.uk/ (accessed on 13 November 2012).
12. Chandra, A.; Acosta, J.; Stern, S.; Uscher-Pines, L.; Williams, M.V.; Yeung, D.; Garnett, J.; Meredith, L.S. *Building Community Resilience to Disasters: A Way Forward to Enhance National Health Security*; RAND Corporation: Santa Monica, CA, USA, 2010.
13. Christian, M.D.; Kollek, D.; Schwartz, B. Emergency preparedness: What every health care worker needs to know. *Can. J. Emerg. Med.* **2005**, *7*, 330–337.

14. Frumkin, H.; Hess, J.; Luber, G.; Malilay, J.; McGeehin, M. Climate change: The public health response. *Am. J. Public Health* **2007**, *98*, 435–445.
15. Gaiser, R.R.; Cheek, T.G.; Gutsche, B.B. Glass recycling in the labour suite is environmentally sound and economical. *Br. J. Anaesth.* **2004**, *92*, 584–586.
16. Getz, L. The state of hospital nutrition. *Today's Dietitian* **2012**, *14*, 44.
17. Gilmour, D. Greening perioperative care. *J. Perioper. Pract.* **2009**, *19*, 268.
18. Greer, A.; Ng, V.; Fisman, D. Climate change and infectious diseases in North America: The road ahead. *Can. Med. Assoc. J.* **2008**, *178*, 715–722.
19. Haines, A.; Kovats, R.S.; Campbell-Lendrum, D.; Corvalan C. Climate change and human health: Impacts, vulnerability and public health. *Public Health* **2005**, *120*, 585–596.
20. Health Canada. Health Facilities Preparation for Extreme Heat: Recommendations for Retirement and Care Facility Managers. Available online: http://www.hc-sc.gc.ca/ewh-semt/pubs/climat/health_facilit-instal_sante/index-eng.php (accessed on 4 October 2012).
21. Health Canada. Emergency Management: Taking a health perspective. *Health Policy Res. Bull.* **2009**. Available online: http://www.hc-sc.gc.ca/sr-sr/pubs/hpr-rpms/bull/2009-emergency-urgence/index-eng.php (accessed on 11 September 2012).
22. Hebert, E. Embracing sustainability to save costs, environment. *Healthc. Exec.* **2008**, *23*, 40–41.
23. Hooke, W.H.; Rogers, P.G. *Public Health Risks of Disasters, Communication, Infrastructure, and Preparedness Workshop Summary*; The National Academies Press: Washington, DC, USA, 2005.
24. Jameton, A.; Pierce, J. Environment and health: Sustainable health care and emerging ethical responsibilities. *Can. Med. Assoc. J.* **2001**, *164*, 365–369.
25. Joint Commission on Accreditation of Healthcare Organizations (JCAHO). 2006 Hospital Accreditation Standards for Emergency Management Planning, Emergency Management Drills, Infection Control, Disaster Privileges. Available online: https://www.efilmgroup.com/efg/blurbimage/JCAHOStdforEmergency.pdf (accessed on 27 October 2012).
26. Kelly, A. Water consumption cut and money saved. *Health Estate* **2012**, *66*, 26–27.
27. Kollek, D.; Karwowska, A. Populations at risk—Paediatrics. *Radiat. Prot. Dosim.* **2009**, *134*, 191–192.
28. Kouadio, I.; Aljunid, S.; Kamigaki, T.; Hammad, K.; Oshitani, H. Infectious diseases following natural disasters: Prevention and control measures. *Expert Rev. Anti-Infect. Ther.* **2012**, *10*, 95–104.
29. Kozicki, Z.A.; Baiyasi-Kozicki, S.J.S.; Thymes, N. Waterborne pathogen treatment of surgical water in U.S. hospitals: Negative implications for vulnerable patient populations (elderly, immune-suppressed, and pediatric). *J. Am. Geriatr. Soc.* **2012**, *60*, 1178–1179.
30. Kulick, M. Healthy Food, Healthy Hospitals, Healthy Communities. Available online: http://www.noharm.org/lib/downloads/food/Healthy_Food_Hosp_Comm.pdf (accessed on 17 November 2012).
31. Kunzli, N. The public health relevance of air pollution abatement. *Eur. Respir. J.* **2002**, *20*, 198–209.
32. Kwakye, G.; Brat, G.; Makary, M. Green surgical practices for healthcare. *Arch. Surg.* **2011**, *146*, 131–136.
33. Lewis, C. Going green in hospital finance. *Healthc. Financ. Manag.* **2008**, *62*, 24–25.
34. Minnesota Department of Health Minnesota Long-term Care Preparedness Toolkit. Available online: http://www.health.state.mn.us/ (accessed on 27 October 2012).

35. Mason, C. Healthy people, places and transport. *Health Promot. J. Aust.* **2000**, *92*, 190–196.
36. McGain, F.; Hendel, S.A.; Story, D.A. An audit of potentially recyclable waste from anaesthetic practice. *Anaesth. Intensiv. Care* **2009**, *37*, 820–823.
37. Meno, K. In Case of Emergency—Hospital Staff Need Proper Preparation for Potential Disasters. *Today's Dietitian*. Available online: http://www.todaysdietitian.com/ (accessed on 17 November 2012).
38. Moore, K.M.; Bronwen, L.E.; McGuiness, D. Implementation of an automated, real-time public health surveillance system linking emergency departments and health units: Rationale and methodology. *Can. J. Emerg. Med.* **2008**, *10*, 114–119.
39. National Health Service (NHS), Sustainable Development Unit. The greener your trust, the greater your benefit. *Health Serv. J.* **2010**, *120*, 18–19.
40. Nova Scotia Government (NSG). Toward a Greener Future: Nova Scotia's Climate Change Action Plan. Nova Scotia Department of Environment. Available online: www.climatechange.gov.ns.ca (accessed on 27 August 2012).
41. Ontario Health Association (OHA). OHA Emergency Management Toolkit: Developing a Sustainable Emergency Management Program for Hospitals. Available online: www.oha.com (accessed on 27 October 2012).
42. Pan American Health Association (PAHO). Principles of Disaster Mitigation in Health Facilities. Available online: http://www.paho.org/english/ped/fundaeng.htm (accessed on 27 August 2012).
43. Parham, J. Path to green: Practice improvements in the OR. *AORN J.* **2011**, *93*, 792–795.
44. Pate, M.F. It is easy being green: Greening the pediatric intensive care unit. *Am. Assoc. Crit. Care Nurses Adv. Crit. Care* **2012**, *23*, 18–23.
45. Rhea, S. Power plays: Healthcare providers look to green energy suppliers, alternative purchasing suppliers to help control costs. *Mod. Healthc.***2009**, *39*, 28–30.
46. Simpson, R.L. Environmental health—Do no harm: Technology creates "green" opportunity for nursing. *Nurs. Adm. Q.* **2010**, *34*, 353–355.
47. Slater, J. Community Food Security: Position of Dietitians of Canada. Available online: http://www.dietitians.ca/Downloadable-Content/Public/cfs-position-paper.aspx (accessed on 17 November 2012).
48. Strashok, C.; Dale, A.; Herbert, Y.; Foon, R. Greening Canadian Hospitals. Available online: http://www.sustainabilitysolutions.ca/sites/default/files/Greening%20Canadian%20Hospitals.pdf (accessed on 27 September 2012).
49. Sulzback, M. What's driving Twin Cities air quality? *Minn. Med.* **2006**, *89*, 36–39.
50. Topf, M. Psychological explanations and interventions for indifference to greening hospitals. *Health Care Manag. Rev.* **2005**, *30*, 2–8.
51. Toronto Public Health. List of Reportable Diseases in Ontario. Available online: http://www.toronto.ca/health/cdc/communicable_disease_surveillance/list_disease.htm (accessed on 23 September 2012).
52. Ueckermann, J. Green Initiatives in Hospitals in Ontario: Is There a Business Case? Available online: http://www.greenhealthcare.ca/component/content/article/221-news8 (accessed on 27 August 2012).

53. University of British Columbia. Outdoor Air Quality and Health and the Air Quality Health Index. E-Learning Course University of British Columbia School of Population and Public Health. Available online: http://spph.ubc.ca/continuing-education/current-courses/outdoor-air-quality/ (accessed on 28 October 2012).
54. United States Centre for Disease Control. Diagnosis and Management of Food-Borne Illnesses: A Primer for Physicians and Other Healthcare Professionals. Available online: http://www.cdc.gov/mmwr/preview/mmwrhtml/rr5304a1.htm (accessed on 27 October 2012).
55. United States Center for Disease Control (CDC). Emergency Water Supply Planning Guide for Hospitals and Health Care Facilities. Available online: http://www.cdc.gov/healthywater/pdf/ emergency/emergency-water-supply-planning-guide.pdf (accessed on 3 December 2012).
56. Vittori, G. Greening of healthcare: Location and design of facilities are essential to the mission of health. *Mod. Healthc.* **2011**. Available online: http://www.modernhealthcare.com/ (accessed on 17 October 2012).
57. Wilson, E.; Garcia, A.C. Going green in food services: Can healthcare adopt environmentally friendly practices? *Can. J. Dietet. Pract. Res.* **2011**, *72*, 43–47.
58. World Health Organization (WHO). Safe Hospitals in Emergencies and Disasters: Structural, Non-structural and Functional Indicators. Available online: http://apps.who.int/bookorders/ anglais/detart1.jsp?codlan=1&codcol=52&codcch=144 (accessed on 27 October 2012).
59. World Health Organization. Prevention of Hospital Acquired Infections – A Practical Guide. Available online: http://www.who.int/csr/resources/publications/whocdscsreph200212.pdf (accessed on 27 October 2012).
60. World Health Organization. Foodborne Disease Outbreaks: Guidelines for Investigation and Control; Surveillance to Detect Food-Borne Disease Outbreaks. Available online: http://www.who.int/foodsafety/publications/foodborne_disease/outbreak_guidelines.pdf (accessed on 24 October 2012).
61. World Health Organization (WHO). Save Lives: Make Hospitals Safe in Emergencies. Available online: http://www.who.int/world-health-day/2009/en/index.html (accessed on 27 October 2012).
62. World Health Organization (WHO). Healthy Hospital, Healthy Hospitals, Healthy People: Addressing Climate Change in Health Care Settings. Available online: http://www.who.int/ globalchange/publications/healthcare_settings/en/index.html (accessed on 27 August 2012).
63. World Health Organization. Strengthening Health Systems' Response to Crises: Towards a New Focus on Disaster Preparedness. Available online: http://www.euro.who.int/__data/assets/ pdf_file/0004/79006/E87920.pdf (accessed on 27 October 2012).
64. Yellowlees, P.; Chorba, K.; Parish, M.B.; Wynn-Jones, H.; Nafiz, N. Telemedicine can make healthcare greener. *Telemed. J. E-Health* **2010**, *16*, 229–232.

© 2014 by the authors; licensee MDPI, Basel, Switzerland. This article is an open access article distributed under the terms and conditions of the Creative Commons Attribution license (http://creativecommons.org/licenses/by/4.0/).
